# The Future of Positive Psychology and Disability

**DOI:** 10.3389/fpsyg.2021.790506

**Published:** 2021-12-09

**Authors:** Michael L. Wehmeyer

**Affiliations:** Department of Special Education, School of Education and Human Sciences, University of Kansas, Lawrence, KS, United States

**Keywords:** disability, strength-based, self-determination, quality of life, optimal human functioning

## Abstract

For much of the history of the application of psychology to disability, the research and clinical focus of the field was deficits-oriented: documenting what people with disability could not do, proposing theories of why they could not do these things, creating measures to assess this incapacity and incompetence, and building interventions and treatments predicated on disease and pathology. It has been only in the last few decades that conceptualizations of disability allowed for consideration of strengths and positive attributes along with the presence of disability and only in the past two decades that a positive psychology of disability has emerged. This article will briefly summarize the factors that led to the emergence of a focus on the positive psychology of disability and a strength-based approach in the field, examine the state of knowledge and practice as it pertains to the positive psychology of disability, and will examine challenges that serve as barriers to progress in this area and opportunities for advancement. Among these is examining how “optimal human functioning” can be understood in ways that includes, and not excludes, people with disability. The importance of shifting the disability research and practice focus to emphasize flourishing, well-being, and self-determination of and for people with disability will be discussed, as well as the necessity for the field of positive psychology to more aggressively reach out to include people with disability among those populations whom the field values and includes.

## Introduction

For much of the history of psychology and before that of medicine and psychiatry, disability has been conceptualized within the context of diseases and disorders (Wehmeyer, 2013a). Societal responses to disability, whether in education, residential services, or rehabilitation services, emphasized segregation and homogeneous grouping in the name of being better able to deliver supposedly critical specialized, and often highly medicalized, services, and treatments. From institutions for people with epilepsy or intellectual disability to center- and facility-based recreation and leisure services for people with physical disabilities, to separate schools for students with emotional or behavioral disorders, to group homes and sheltered workshops for people with intellectual and developmental disabilities, the systems developed to provide services to people with disabilities were segregated and based on conceptualizations of disability as pathology, disease, or deficit (Smith and Wehmeyer, 2012).

Through the latter part of the 20th century and into the 21st century, multiple factors and events converged and coalesced to create opportunities for people with disabilities to break free from the stereotypes, biases, and discrimination that limited their autonomy and self-determination. In the next section of this article, I briefly describe these multiple factors and events and, following, use that as a base for considering the future of positive psychology and disability.

## From Pathology to Positive Psychology

The transformation of disability from being viewed as a pathology to consideration within positive psychology began worldwide post-WWII with the demands of parents and family members for alternatives other than institutionalization and isolation (Abeson and Davis, 2000). This parental movement resulted in legislation and civil protections that, often for the first time, ensured access to education, rehabilitation, and the community for many people with disabilities (Thompson et al., 2017). According to Thompson et al. (2017), the field had moved from a medical-institutional paradigm driving services to the normalization-community services paradigm. Services in this era were driven by a principle of normalization that emphasized community inclusion and normal routines of the day (Nirje, 1969). Unfortunately, while many people moved from large congregate settings to smaller, community-based settings and many students with disabilities gained access to education, these were still primarily congregate or segregated in nature, and services were still driven by a pathology perspective of disability. In the latter two decades of the 20th century, however, the medical field recognized that viewing chronic and long-term health conditions, including disability, through a pathology lens had limited utility. Conceptualizations that emphasized more than just pathology or disorder were introduced, culminating in 2001 in the World Health Organization’s International Classification of Functioning, Disability, and Health (ICF; World Health Organization, 2001). The ICF conceptualized disability not as a disease or a disorder, but as the outcome of the interaction among health conditions (impairments), personal factors, and environmental factors. The ICF introduced so-called person-environment fit or social-ecological models to the disability context, and disability was conceptualized not as an internal pathology within the person, but as resulting from the interaction between what one can do and what the environment or context supports. The result, according to Thompson et al. (2017) was that the field moved toward a supports paradigm where emphasis was on enhancing personal capacity, modifying environments and contexts to promote participation, and providing supports that enable people to function successfully in typical environments and activities.

At the same time that the ICF was introduced, the field of positive psychology was emerging with its emphasis on positive individual traits, personal strengths, and well-being (Seligman and Csikszentmihalyi, 2000; Peterson, 2009). The ICF provided, for the first time, a language for a strength-based approach to disability, which aligned with the intent and objectives of the positive psychology movement (Wehmeyer, 2013b). In the past two decades, some progress has been made in applying positive psychology and strength-based approaches to the disability context and that is briefly covered in the next section.

### State of Knowledge and Practice as it Pertains to the Positive Psychology of Disability

The publication of the *Oxford Handbook of Positive Psychology and Disability* in 2013 (Wehmeyer, 2013b) provided the first comprehensive examination of the application of positive psychology to the disability context. In that volume, Shogren (2013) provided an historical analysis of the state of research with regard to the emphasis on positive psychological constructs in the disability literature and of the disability focus in the positive psychology literature. In a nutshell, what Shogren et al. (2006b) found was that in a three-decade analysis of journals in the field of intellectual disability, there was a steady positive trend of the percentage of papers addressing constructs in positive psychology or strength-based approaches but that the focus on disability in positive psychology journals was minimal and limited mainly to research on chronic illnesses.

Even though there were clear limitations to the literature, there were positive psychological constructs that had a rich history in the disability context, in particular quality of life and self-determination, and there were a number of such constructs that had an emerging focus in the disability context, including mindfulness and character strengths. Within the disability context, the quality of life construct has provided “an outcomes-based evaluation framework associated with specific life domains that enable both the consideration of personally valued life outcomes and the design of large systems of supports” (Wehmeyer, 2020, p. 7). In fact, as conceptualized by Schalock (1996), quality of life is a meta-construct, incorporating and defined by multiple positive psychological constructs, including the core dimensions of self-determination and financial, physical, and mental well-being, as well as emphasizing outcomes of social inclusion, personal development, and interpersonal relationships. Quality of life, argued Schalock and Verdugo (2013), has provided a framework for a disability service delivery system that is “based on the values of dignity, equality, empowerment, self-determination, non-discrimination, and inclusion” (p. 46). Quality of life is not a “thing” that people have but is a multidimensional construct that provides a means to design and evaluate supports for people in service systems (Schalock, 1996).

Another positive psychological construct that has extensive application to the disability context is self-determination. Research over the past three decades has been driven by a theoretical framework, Causal Agency Theory, which provides a theory of the development of self-determination (Wehmeyer, 1992, 2001; Shogren et al., 2015a; Wehmeyer et al., 2017). This theoretical perspective has drawn from Self-Determination Theory (SDT; Ryan and Deci, 2000, 2017), but unlike SDT, which is a meta-theory of motivation, Causal Agency theory attempts to understand how people become self-determined and, as a result, facilitate the design and evaluation of interventions and supports that support this development. Causal agency theory defined self-determination as:

… a dispositional characteristic manifested as acting as the causal agent in one’s life. Self-determined people (i.e., causal agents) act in service to freely chosen goals. Self-determined actions function to enable a person to be the causal agent in his or her life (Shogren et al., 2015a, p. 258).

Acting in a self-determined manner implies that people make or cause things to happen in their own lives, rather than someone or something else making or causing them to act in other ways. Self-determined action refers to action that is volitional and agentic (Shogren et al., 2015a). There is a rich literature driven by Causal Agency Theory documenting that young people with disability who are more self-determined achieve better school and post-school outcomes (Shogren et al., 2012, 2015b), that youth and adults with disability who are more self-determined have more positive quality of life and life satisfaction (Wehmeyer and Schwartz, 1998; Lachapelle et al., 2005; Shogren et al., 2006a), and that people with disabilities can become more self-determined when provided opportunities to learn and put in practice skills related to causal agency, they do become more self-determined (Wehmeyer et al., 2012a,b).

As noted, there are several other positive psychology constructs that are receiving increased attention in disability research, including mindfulness and character strengths (Shogren et al., 2017). In the field of education, a focus on positive education is emerging as it relates to the education of learners with disabilities (Kern and Wehmeyer, 2021). Hope, optimism, resilience, and coping are also topics that have an emerging literature base, the latter two particularly in the fields of social and rehabilitation psychology (Dunn, 2019; Wehmeyer and Dunn, 2021). Finally, issues pertaining to disability identity have played an increasing role in efforts to move toward strength-based approaches to disability (Andrews and Forber-Pratt, 2021).

### Challenges That Serve as Barriers to Progress in the Application of Positive Psychology in the Disability Context and Opportunities for Advancement

There are a number of challenges that serve as barriers to progress in the application of positive psychology to the disability context, from the siloed nature of academic and clinical disciplines to limited funding for research in this area. But the overarching barrier to progress in this area is that how disability is understood in society, in general, and in psychology and related disciplines, in particular, remains mired in pathology-based conceptualizations. Positive psychology is, fundamentally, the study of flourishing, optimal human functioning, and well-being (Seligman and Csikszentmihalyi, 2000). Far too many people, both in the public and in psychology, continue to perceive disability as a burden to be borne or as an illness or disease to be fixed or cured. People with disabilities continue to experience stigma associated with others’ views about and understandings of disability and continue to pity or, sometimes, fear people with disability. The inevitable results are not only discrimination and marginalization, but also the inability of others to see that people with disability possess strengths and can, indeed, flourish and experience well-being. Put bluntly, the public and the field of psychology too often do not understand how “optimal human functioning” can apply to the life experiences of a person with a disability.

## Discussion

As documented in the prior sections, there has been progress in shifting the disability research and practice focus to emphasize flourishing, well-being, and self-determination, yet barriers related to attitudes about and understandings of disability itself remain. In the closing section, I propose a number of actions that need to occur to increase a focus on positive psychology in the disability context.

### The Future of Research and Practice in the Application of Positive Psychology to the Disability Context

Although there are actions that are needed in both positive psychology and disability-related fields to ensure that the progress in applying positive psychological constructs to the disability context continues, the following action items focus exclusively on the field of positive psychology.

#### Researchers and Practitioners in Positive Psychology Must More Aggressively Reach Out to Include People With Disability

As noted previously, although the application of positive psychological constructs in disability-related research has increased steadily, the same does not seem to be as true for research in positive psychology. To the degree that research-derived interventions and treatments drive practice, the relative scarcity of such disability-focused research in positive psychology is an area for growth. There are some disciplines within psychology, notably rehabilitation psychology, that have begun to embrace a positive approach, but the opportunity exists for positive psychologists to lead into the future by embracing disability as part of the continuum of human experiences and by showing how people with disabilities can be supported to flourish.

#### Psychologists With Disabilities Need to Have an Active Voice in Shaping the Field of Positive Psychology

One of the ways in which positive psychology can increase its focus on the disability experience is by training, hiring, and supporting psychologists with disabilities to enter and stay in the field. The presence of psychologists with personal experience with disability will energize the discussion about what it means to flourish in a diverse society and ultimately bring insights about the human experience and condition that will otherwise be overlooked or ignored.

#### Models of Disability That Emphasize Disability as Part of the Human Experience Must Drive Research and Practice

The World Health Organization’s shift toward social-ecological or person-environment fit models of conceptualizing disability dates back to the early 1980s, and the disability community’s emphasis on understanding disability within a social context dates back to at least the same era. These ways of understanding and conceptualizing disability provide a language for strength-based approaches to supporting people with disabilities to live full, rich lives in their communities. By positive psychology embracing these understandings and practices, the field can move forward to consider how flourishing and optimal human development co-occur with the experience of disability.

The field of positive psychology is in its nascence. The fact that there is already a focus on the application of positive psychological constructs to the disability context gives hope that the discipline can grow with a disability focus as part of its core. As the editor of the *Handbook of Positive Psychology and Disability*, published in 2013, I have seen considerable growth in the disability literature pertaining to positive psychological constructs. There are obvious areas of potential growth and expansion for the next decade. For one, positive psychological constructs for which research and practice in the disability context is just emerging have potential for growth. These include constructs, such as hope, optimism, character strengths, mindfulness, and resilience. As a disability culture and identity focus spreads there will be greater opportunities to explore the relationships between a positive disability identity and positive psychology. Further, as described previously, there is a long history of research in the disability context on quality of life. But this work focuses mainly at organizations and the services they provide. Research on flourishing and well-being among people with disability is an area of potential growth and benefit. The field of positive education is a growing subdiscipline in positive psychology and there is potential for applying topics, such as youth development, creativity, self-determined learning, and positive emotions in the disability context for research and practice. Although there is a well-established research and practice base focusing on self-determination in the disability context, agency is a central theme in positive psychology, and there is a need for an expanded focus on motivation and agency in the disability context. Finally, although there is a rich research and practice base in assessment pertaining to positive psychology, too often such tools are not normed with nor developed with people with disabilities, so there is opportunity to increase the number of assessments developed to include people with disability and to validate existing measures with people with disability.

In summary, the progress in applying positive psychology to the disability context in the past decade has been encouraging, and by embracing some of the action items identified in the article, there are rich and varied opportunities available that will keep this progress moving into a future that includes and embraces people with disabilities as important to the field of positive psychology.

## Author Contributions

The author confirms being the sole contributor of this work and has approved it for publication.

## Conflict of Interest

The author declares that this article was written in the absence of any commercial or financial relationships that could be construed as a potential conflict of interest.

## Publisher’s Note

All claims expressed in this article are solely those of the authors and do not necessarily represent those of their affiliated organizations, or those of the publisher, the editors and the reviewers. Any product that may be evaluated in this article, or claim that may be made by its manufacturer, is not guaranteed or endorsed by the publisher.
